# Going Online?—Can Online Exercise Classes during COVID-19-Related Lockdowns Replace in-Person Offers?

**DOI:** 10.3390/ijerph19041942

**Published:** 2022-02-09

**Authors:** Eszter Füzéki, Jan Schröder, Rüdiger Reer, David A. Groneberg, Winfried Banzer

**Affiliations:** 1 Division of Preventive and Sports Medicine, Institute of Occupational, Social and Environmental Medicine, Goethe-University Frankfurt, Theodor-Stern-Kai 7, 60590 Frankfurt, Germany; groneberg@med.uni-frankfurt.de (D.A.G.); banzer@med.uni-frankfurt.de (W.B.); 2 Department of Sports Medicine, Faculty for Psychology and Human Movement Science, Institute for Human Movement Science, University of Hamburg, Turmweg 2, 20148 Hamburg, Germany; jan.schroeder@uni-hamburg.de (J.S.); ruediger.reer@uni-hamburg.de (R.R.)

**Keywords:** internet, confinement, coronavirus, physical activity

## Abstract

Germany experienced a 6-month second lockdown (November 2020–April 2021) during the COVID-19 pandemic, which included the closure of all physical activity (PA) facilities. The use of online exercise classes (OECs) was promoted by public health and exercise organizations. Using the present cross-sectional online survey, we assess the use of and opinion towards OECs in Germany during the second lockdown. We used contingency tables and the Chi^2^ test to calculate the frequency of awareness and use of OECs according to PA status, well-being and demographic data, and conducted a binary logistic regression with OEC awareness or use and dichotomized independent predictors. The associations between opinion and activity status, frequency of use, educational attainment, age and body mass index were calculated using Spearman correlations. A total of 993 datasets were analyzed in detail. Of the 785 (79.1%) participants reporting awareness of OECs, 536 tried them, and 262, 188 and 85 used them <1 per week, 1–2 per week and ≥3 per week, respectively. The users were typically active, female participants with poorer mental well-being. The opinions towards OECs varied according to participant characteristics, such as activity status, BMI and age. Overall, regular OEC use was quite limited, and, as such, cannot replace in-person exercise opportunities. Keeping physical activity facilities open and safe must be prioritized in the ongoing pandemic.

## 1. Introduction

The COVID-19 disease, which was first documented in late 2019 in China, reached pandemic dimensions and was declared as such by the World Health Organization (WHO) in mid-March 2020 [1]. As early as May 2020, Kissler and colleagues forecasted that “recurrent wintertime outbreaks are likely to occur in coming years” [2]. Indeed, to date, the pandemic has come in distinct waves in many countries, including Germany [3]. Whereas in some countries, e.g., Italy, the first wave was devastating, and the second somewhat milder [4], Germany experienced a relatively short and less severe first wave [3]. The second wave (from September 2020 until February 2021), however, lasted much longer and had markedly higher case numbers and fatalities [3]. The vaccination process started in the final days of December 2020, but rates remained too low for many months to effectively curb the infection [5]. In response, the federal government reinstated traditional public health measures limiting personal contact in many areas of daily life. A partial national lockdown was implemented on 2 November 2020, and was strengthened as of 16 December 2020 [6]. By virtue of the lockdown, all physical activity (PA) facilities, such as sports and fitness clubs and swimming pools, were closed. In late April 2021, a federally unified regulation was adopted, which coupled all future restrictive and/or opening measures to regional incidence values [7]. As the incidence values showed differences within the country, there were also some regional differences in the reopening of PA facilities. Since no widespread opening was possible before April 2021, PA facilities remained closed for about six months.

Social distancing and the restriction measures led to the reduction in infection and mortality rates, but at the same time had serious detrimental repercussions, some of which were already predicted early on in the pandemic [2]. To date, low PA levels have declined further in many countries, including Germany [8,9]. Parallel to this, high levels of overweight and obesity have increased further [10], and mental health has seriously deteriorated [11,12]. The reduction in PA levels is alarming, since—beyond the well documented effects on the risk and progression of chronic diseases—the body of evidence on the protective effects against severe COVID-19 disease and death is increasing [13,14].

During the lockdown period, governmental, professional PA and health organizations have taken efforts to promote PA in the general population [15,16]. “Online exercise classes” (OECs) and “workout videos” have been explicitly recommended by the World Health Organization (WHO) Regional Office for Europe and the Centers for Disease Control and Prevention [15,16]. In Germany, many community-based sports clubs provided alternative exercise opportunities to compensate for the loss of in-person courses [17,18].

OECs have a seeming appeal, especially during the pandemic. They are time flexible, in many cases free of charge, and can be practiced without any infection risk. We conducted a survey during the first lockdown in Germany, during which also all PA facilities were closed, which showed that while almost 70% of respondents reported an awareness of OECs, few (12% of those reporting awareness; 9% of all respondents) were regular (≥3 per week) users [19]. Respondents’ perceptions of different aspects of OECs, such as time flexibility, lack of supervision and lack of interaction with other exercisers, were different according to characteristics, such as age, body mass index (BMI) and educational attainment [19].

In the spring of 2021, we repeated our survey to assess the extent to which OECs are known and used in Germany, and the opinions of users concerning them. We hypothesized that (1) a large majority of respondents were aware of OECs, (2) the regular use of OECs was not widespread (3) and that the use of OECs showed distinct patterns in participant characteristics. Finally, we aim to provide an exploratory analysis regarding users’ opinions on OECs.

## 2. Materials and Methods

### 2.1. Study Design and Recruitment

We repeated our previous study with the modification that we included the assessment of well-being [19]. We used the SoSci Survey tool (SoSci Survey GmbH, Munich, Germany, https://www.soscisurvey.de/) (accessed on 8 April–2 July 2021) to conduct this cross-sectional anonymous online survey in the German general population. Ethical approval for this study was obtained from Goethe University, Frankfurt (reference number 2020-18). We applied the snowball sampling method to recruit the participants. We disseminated the link to the survey together with a description of the study’s aims and information on the data protection in our professional and personal networks. The recipients of these e-mails were invited to fill out the questionnaire and distribute the link in their respective networks and/or publish it on their websites, newsletter and social media channels. Participants provided informed consent. The identity of the respondents was not traceable. The survey link was live from 8 April–2 July 2021.

### 2.2. Questionnaire

The questionnaire contained three major parts: one on habitual PA, one on well-being and one on the use of and opinion towards OECs [12]. Data on habitual PA and well-being can be found elsewhere [12]; here, we report on the OECs. We used the respective questions from the European Health Interview Survey (EHIS wave 2) [20] in the official German translation to assess the anthropometric data, such as the height and weight (BM1–2), and PA (PE1–PE8). PA data was assessed via the EHIS Physical Activity Questionnaire (EHIS-PAQ), which is a validated instrument to measure PA according to current PA recommendations [21]. We used the World Health Organization Well-Being Index (WHO-5) to measure well-being. The WHO-5 is a validated five-item global rating scale, which can be used as a screening tool for depression with a cut-off value of 50 out of the maximal 100 [22]. The instrument contains five positively phrased items to which respondents provide answers on a five-point Likert scale (0 to 5 points; 0 = never, 1 = sometimes, 2 = less than half of the time, 3 = more than half of the time and 4 = mostly, 5 = always). Reference values are available for a large number of countries, including Germany [22].

After indicating whether or not they were aware of OECs, the participants who answered with “affirmative”, were asked whether they had already used such offers. Those who reported having used OECs specified the frequency of use per week. The users’ opinions were assessed using a four-point Likert scale (1 = strongly agree, 2 = agree, 3 = disagree and 4 = strongly disagree) for five statements on OECs (OECs allow for time flexibility; by using OECs, I miss the interaction with other exercisers; with OECs, I motivate myself less successfully than with in-person classes; with OECs, I miss the individual guidance of the trainer; and OECs are boring).

The participants’ highest educational attainments were recorded using the International Standard Classification of Education (ISCED 2011) [23].

### 2.3. Data Processing and Statistical Analysis

We processed and scored PA data according to the official EHIS-PAQ scoring protocol, and categorized participants as active (at least 150 total minutes of sports, fitness and leisure time physical activity (LTPA) in at least 10 min bouts per week, plus muscle strengthening activities for at least two days a week) or inactive in terms of the current WHO PA guidelines [24]. The BMI was calculated using self-reported body weight and height.

For detailed analyses, we dichotomized predicting or confounding variables for OEC awareness or use, such as sex (female vs. male), age (older vs. younger, age ≥ 50 years vs. age < 50 years), BMI (obese vs. non-obese, BMI ≥ 30 kg/m^2^ vs. BMI < 30 kg/m^2^), well-being (depressive vs. non-depressive, WHO-5 < 50 points vs. WHO-5 ≥ 50 points), and educational level (academic vs. non-academic, at least bachelor degree vs. primary, secondary or tertiary school education level), as well as PA (active vs. inactive).

We calculated the frequencies (categorical variables) and mean with standard deviation (SD) (scaled parameters) for the basic description. We conducted a binary logistic regression (fixed effects) with OEC awareness or use as dependent variables and dichotomized independent predictors (sex, age, BMI, educational level, PA and well-being). Contingency tables and the Chi^2^ test with Cramer’s V as an effect size measure were used to calculate the frequency distribution of awareness and use of OECs according to confounding variables. The association between the level of agreement to OEC opinion statements and PA status (active vs. inactive) and sex (male vs. female), as well as the scaled variables frequency of OEC use, well-being, educational attainment, age and BMI was tested using the Spearman correlation coefficient (rho). 

We used IBM SPSS software, V.22 (IBM, Armonk, VA, USA), to compute all statistical analyses. The significance level was set at *p* ≤ 0.05.

## 3. Results

### 3.1. Sample Characteristics 

As a result of missing data necessary for the detailed analyses (age, sex, BMI), 12 datasets had to be removed. The remaining 993 datasets (n = 708; 71.3% females, n = 285; 28.7% males) were analyzed in detail. The respondents’ ages were 45.8 ± 14.7 years and they had a BMI of 24.9 ± 5.1 kg/m^2^, with 1.8%, 59.0%, 26.8% and 11.9% of them being categorized as underweight (BMI < 18.5), normal weight (BMI 18.5–24.9), overweight (BMI 25–29.9) and obese (BMI ≥ 30), respectively. The study participants were predominantly (69.2%) well-educated (Bachelor, Master, Doctoral Degrees) and none of them had only a primary education.

The sample characteristics, displayed as the frequencies of dichotomized variables for sex, age, BMI, educational level, PA level, well-being, awareness and use of OECs, are presented in Table 1.

More than half of all the respondents (n = 553 or 55.7%) engaged in ≥150 min/week endurance-type PA, and almost 4 in 10 (n = 386 or 38.9%) in muscle strengthening ≥2 days/week. Compliance with current PA recommendations (both endurance and muscle strengthening) was 29.4% (n = 292).

During lockdown, psychological well-being showed an average value of 51.5 ± 21.3 points (n = 991). Almost half of all the respondents (46.8%) were below the cut-off score of ≤50 points indicating depressive symptoms, while 528 (53.2%) showed no symptoms of depression. 

An awareness of OEC was reported by n = 785 (79.1%) respondents, and the use of OECs by n = 536 (54.0%), respectively. 

### 3.2. Awareness of Online Exercise Classes 

Compared to males, a significantly lower rate of female respondents reported not being aware of OECs (*p* < 0.001). Respondents reporting a lack of awareness of OECs were more likely to be 50 years or older (*p* < 0.001), to have a BMI of 30 kg/m^2^ or above (*p* = 0.007) and to be classified as inactive according to WHO recommendations (*p* < 0.001). 

We found no significant differences in psychological well-being (*p* = 0.179), and in educational attainment (*p* = 0.510) between those aware of OECs (n = 785) and those not aware of OECs (n = 208), cf. also Table 2.

The only significant predictor in our binary regression model of the dichotomized dependent variables in order to explain OEC awareness was sex (*p* < 0.001), demonstrating a β coefficient of 1.430. Females had a more than four-fold higher chance (417.9%) of being aware of OECs, cf. Table 3.

### 3.3. Use of Online Exercise Classes

Of the subsample reporting an awareness of OECs (n = 785), 536 tried them, and 262, 188 and 85 used them <1 per week, 1–2 per week and ≥3 per week, respectively, 1 respondent failed to indicate the frequency of use, cf. also Table 1.

Compared to the males, a significantly lower rate of female respondents reported no use of OECs (*p* < 0.001). Respondents reporting no use of OECs were more likely to be 50 years or older (*p* = 0.048), to have a BMI of 30 kg/m^2^ or above (*p* = 0.012) and to be inactive according to WHO recommendations (*p* < 0.001). 

We found no significant differences in psychological well-being (*p* = 0.658), and in educational attainment (*p* = 0.156) between OEC users (n = 536) and non-users OEC (n = 249), cf. also Table 4.

We found a non-significant association between the PA status (active vs. inactive) and the frequency of use of OECs (X^2^[536, 3] = 6.145, *p* = 0.105, V = 0.11) in users (n = 536), cf. also Table 5.

As significant binary logistic regression predictors of OEC use, we identified the dichotomized dependent variables as sex (*p* < 0.001, β = 1.501, OR = 4.486), PA (*p* < 0.001, β = −0.671, OR = 0.511) and well-being (*p* = 0.028, β = 0.344, OR = 1.410). Females and respondents with a worse well-being (WHO-5 < 50 points) had higher chances (448.6% and 41.0%, respectively) to be users. On the other hand, respondents classified as inactive showed reduced chances (−48.9%) to be users. Age, BMI and education as predictors were not significant in our regression model, cf. Table 6.

### 3.4. Users’ Opinions towards Online Exercise Classes

**Figure 1:**
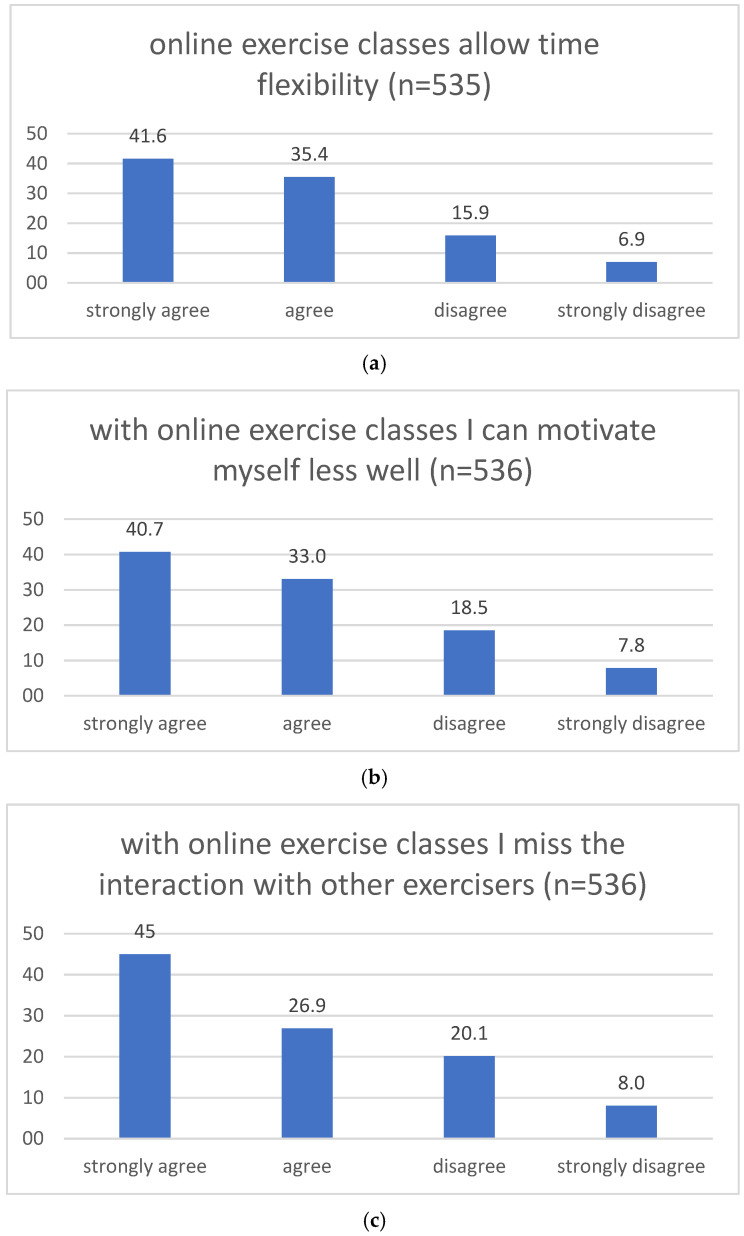

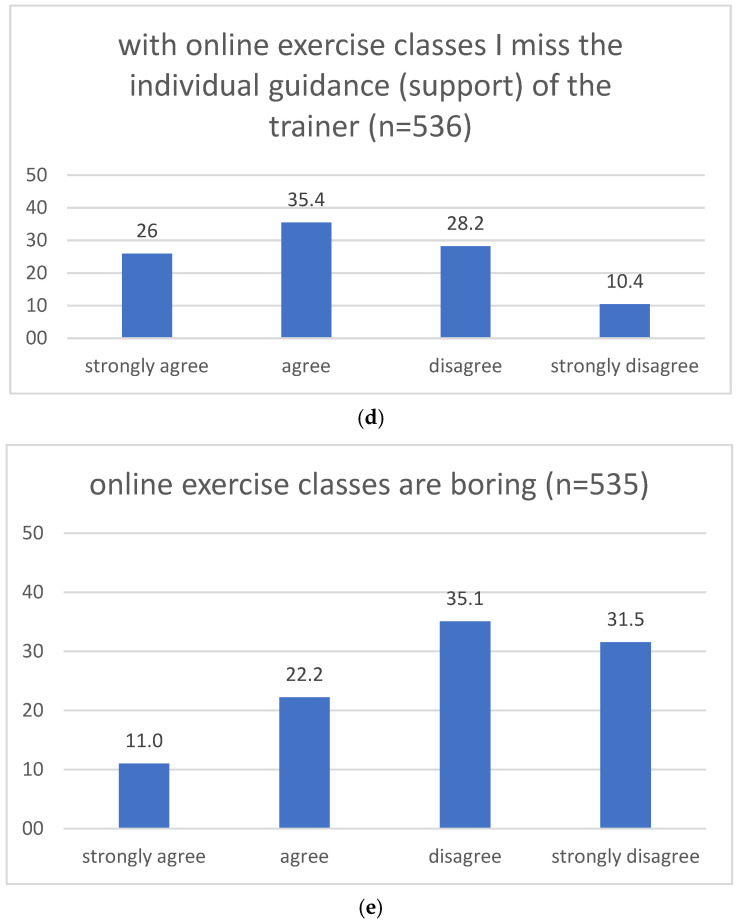
Users’ opinions towards OECs in % (‘time flexibility’ (**a**), ‘self-motivation’ (**b**), ‘social interaction’ (**c**), ‘individual trainer guidance’ (**d**), and ‘boringness’ (**e**)).

The descriptive analysis of users’ opinions towards OECs in % are shown in Figure 1.

Opinions towards OECs showed significant correlations with demographic data, PA, well-being and the frequency of OEC use, cf. Table 7. We found that the respondents who had a higher educational attainment, were physically active in terms of the WHO recommendations, exhibited no depressive symptoms and were frequent OEC users who showed a stronger agreement with the statement on flexible time management. The respondents with depressive symptoms and a lower frequency of use showed a stronger agreement with the statement concerning a lack of interaction with other exercisers. Younger, male, inactive participants, and participants with a lower educational level, depressive symptoms and low frequency of use agreed more to the statement that they found it more difficult to motivate themselves with OECs. A stronger agreement with the statement on missing the instructor’s support was shown by inactive respondents and by those with a higher BMI, depressive symptoms and low frequency of use. A stronger agreement with the statement on boringness was found in male, inactive respondents, and those with depressive symptoms and a low frequency of OEC use.

## 4. Discussion

The main aim of our study was to assess the visibility and use of OECs, as well as users’ opinions on OECs during the second COVID-19-related lockdown in Germany. The results confirmed our hypotheses: OECs were, generally speaking, known to a large majority of all the respondents (almost 80%), yet only 18.9% and 8.5% of all the participants used them 1–2 per week and ≥3 per week, respectively. Awareness was higher in younger responders, and the use of OECs was higher in female participants who were physically active, and showed depressive symptoms. Female respondents were significantly more often aware of and used OECs than males. The frequency of use in the user subsample was not significantly different in active vs. inactive respondents. 

We found a higher awareness (80 vs. 70% respectively) and use rates (24 vs. 18% <1 per week; 19 vs. 12% 1–2 per week) in the present study, compared to our previous survey in the first lockdown, in a sample that was very similar to the present one in terms of sample size, sex distribution, age, BMI, educational attainment and PA levels in the normal condition [19]. Regular use (≥3 per week) was 8.5%, which was similar in both surveys. The reason for the increased awareness might be the time that had elapsed since the beginning of the pandemic. More PA providers might have offered OECs and these might have been more strongly promoted than during the first lockdown, and thus more respondents had the possibility to learn about them. The increased awareness “translated” into an increased rate of low-frequency (<1 per week, 1–2 per week) use; regular use (≥3 per week), however, remained at the same low level.

The comparison of the results with other studies must be observed in light of the considerable heterogeneity in, among other things, the sample characteristics, assessment instruments, lockdown periods and conditions of the different studies. A recent investigation using the daily diary approach also documented the limited use of remote/streaming services and found that only 18% of all PA bouts were facilitated by them in the early phase of the pandemic (April–June 2020) in California and Colorado [25]. Martin and colleagues, however, report significant and large increases in the use of online workouts during the lockdown in recreational sport participants in the United Kingdom, with almost 83% of users during lockdown being non-users pre-lockdown [26]. Similarly, a Belgian study found a significant increase in the use of mediated support, including online videos and online sports classes, during the first lockdown compared to the time before the lockdown [27]. Watching online videos during the lockdown was one of the five predictors of being physically active, according to current guidelines in this report [27].

A representative study from Germany found that about one in five respondents used digital media for sports at least once during the COVID-19 pandemic, but many users returned to in-person exercise as soon as the lockdown was eased [28]. Users were younger, had a higher level of education and higher income [28]. Similar to our findings, the respondents who reported having used digital sport offers tended to be physically active also prior to the pandemic and the lockdowns [28]. 

A qualitative study conducted during the first lockdown in Germany sheds light on this topic from sports club the perspective of personnel (mangers and trainers) [18]. The interviewees’ perceptions were that different target groups, such as children and youth, elderly, individual and team sports players, exhibited differential acceptance and the use of OECs, but no clear pattern was presented [18]. They also expressed certain reservations regarding the extent to which such offers can truly replace in-person courses in the long run [18]. The concerns mentioned included the lack of supervision and guidance, limitations in the case of team sports, and digital and data security issues with certain users [18], some of which are also reflected in our data.

Compared with our previous survey, the agreement (strongly agree and agree) with “negative” OEC-related factors (lack of instructor’s guidance: 61 vs. 54%; lack of interaction with other exercisers: 72 vs. 62%; boringness: 33 vs. 31%; and motivation: 74 vs. 68%) increased, and the agreement with the “positive” aspect (time flexibility: 77 vs. 88%) decreased. This may suggest that the respondents’ perceptions changed from the first to the second lockdown, as if more people saw the glass half empty than half full. The ongoing pandemic and the extensive confinement measures might have worn down people. Indeed, we have documented a dramatic increase in depressive symptoms during the second lockdown in Germany [12]. Mental well-being was also a statistically significant predictor of OEC use in our sample, and the respondents with depressive symptoms seem to be especially impacted by interpersonal characteristics of OECs, such as a lack of interaction with others and the guidance from trainers.

In the present study, almost three quarters of our participants reported that they found it more challenging to motive themselves to exercise using OECs than using regular classes, and this was especially the case in younger males and inactive respondents, and in respondents with depressive symptoms and with a lower educational attainment. The challenge to find motivation for using OECs is echoed in the U.K. study as well [26]. In our study, the lack of support from the instructor was negatively perceived by the participants with a higher BMI and depressive symptoms.

It seems necessary to consider the different waves and phases of the pandemic in a differential manner. While the data on the first wave of the pandemic are abundant [8], much less is known about the effects of the further pandemic course on health-related lifestyles and well-being. The available data, however, indicate that PA levels do not automatically revert to pre-pandemic levels (which was inadequate to begin with) after restrictions have been eased or ended [29,30], and well-being is further reduced with the progression of the pandemic [31]. As predicted early on, the next wave is raging as of November 2021 [32]. A general lockdown, including the closure of all PA facilities, has been reinstalled as of 22 November 2021 in Austria [33] and the German Federal State of Saxony [34]. Restricted access to PA facilities only for people who have been fully vaccinated or have recovered from the disease has been introduced in Germany, if the 7 day COVID-19-related hospitalization rate per 100,000 inhabitants is above 3, which is the case in most parts of Germany in early 2022 [35]. Even without formal lockdowns or restrictions, it is plausible that some people will avoid or limit the use of indoor PA facilities for fear of vaccine breakthrough infections [36]. 

Additionally, there is reason to assume that the current wave of COVID-19 disease in some parts of Europe, including Germany, will not recede for many months to come. A current expert consultation posits the following statement concerning the vaccines: “if not accompanied with comprehensive strategies and public support they alone will not protect from further damaging outbreaks in the coming years” [37]. Against this background, public health and PA professionals should scale up efforts to further increase PA awareness, specifically during the pandemic, promote health-related PA and provide safe and attractive PA and exercise opportunities for different, vulnerable groups. The widespread installation of air cleaners with high-efficiency particulate air filters should be considered in indoor spaces where appropriate outdoor air ventilation is not feasible [38]. The characteristics of OEC users (females and a higher activity level) in the present study seem to suggest a general health consciousness. The regular use of OECs also presupposes a certain level of digital skills and access to high speed Internet as well as appropriate devices, which might not be available at all places and in all person groups [39,40]. Taken together, our results, and those of others, suggest that the use of technology in PA promotion at the population level in pandemic times can only be seen as one of many avenues, but the effects might be limited [25]. In the face of the ongoing pandemic, keeping PA facilities open must be prioritized. 

The present study shows some strengths and limitations. We have a reasonably large sample size, and assessed PA with the validated EHIS PAQ. As a result of the large sample size, we were also able to compute detailed analyses regarding opinions and frequency of use. A limitation of the survey is the use of a non-validated questionnaire to assess opinions, and the predominance of female and well-educated respondents. As such, the results might not be generalizable for other samples. 

## 5. Conclusions

As of November 2021, the COVID-19 pandemic is again gaining momentum in Europe, with the first general lockdowns reinstalled. This development harbors the risk of closure and/or limited use of PA facilities. Data from the first wave underline the immense significance of PA for health and well-being in the pandemic, and the need for concerted measures to promote safe PA. OECs provide a no-risk and in itself easily accessible way to exercise. We found that though they are by now known to the vast majority of people, their appeal is differentially strong and, for some groups of individuals, limited; indeed, regular use was reported only by a small minority of respondents. The lack of social interaction with other exercisers and the lack of guidance from trainers, as well as the difficulty to motivate oneself, might contribute to the limited attractiveness of OECs. Since OECs cannot be regarded as a true replacement for in-person classes, efforts should be intensified to keep in-person exercising possible and safe for all during the ongoing pandemic.

## Figures and Tables

**Table 1:** Sample characteristics (n = 993).

		n	%
Sex	female	708	71.3
	male	285	28.7
Age	≥50 years	460	46.3
	<50 years	533	53.7
BMI	≥30 kg/m^2^	118	11.9
	<30 kg/m^2^	875	88.1
Education	<Bachelor	306	30.8
	≥Bachelor	687	69.2
PA level (WHO)	inactive	701	70.6
	active	292	29.4
Well-being (WHO-5)	<50 points	465	46.8
	≥50 points	528	53.2
OEC awareness	no	208	20.9
	yes	785	79.1
OEC use	no	249	25.1
	yes	536	54.0
Use < 1 per week		262	26.4
Use 1–2 per week		188	18.9
Use ≥ 3 per week		85	8.6
No answer		1	0.1

Abbreviations: BMI = body mass index, PA = physical activity, WHO = World Health Organization and OEC = online exercise class.

**Table 2:** Differences between the respondents being aware (n = 785) and not being aware (n = 208) of OECs.

		OEC Not Aware	OEC Aware	X^2^ _(993, 1)_	*p*-Value	ES
Sex	female	17.2%	82.8%	20.560	<0.001	0.14
	male	30.2%	69.8%			
Age (years)	≥50	27.2%	72.8%	20.070	<0.001	0.14
	<50	15.6%	84.4%			
BMI (kg/m^2^)	≥30	30.5%	69.5%	7.394	0.007	0.09
	<30	19.7%	80.3%			
Education	<Bachelor	22.2%	77.8%	0.435	0.510	0.02
	≥Bachelor	20.4%	79.6%			
PA	inactive	25.4%	74.6%	28.453	<0.001	0.17
	active	10.3%	89.7%			
Well-being (WHO-5 pts.)	<50	22.8%	77.2%	1.806	0.179	0.04
	≥50	19.3%	80.7%			

Abbreviations: OEC = online exercise class, ES = effect size, BMI = body mass index, PA = physical activity and WHO = World Health Organization.

**Table 3:** Binary logistic regression to predict the awareness of online exercise classes.

Dependent:	OEC Awareness	β	*p*-Value	OR	95% CI	
n = 993					Lower	Upper
Sex	female vs. male	1.430	<0.001	4.179	3.188	5.477
Age	50 or older vs. younger	−0.078	0.587	0.925	0.699	1.224
BMI	obese vs. BMI < 30	−0.270	0.239	0.763	0.486	1.197
Education	non-academic vs. academic	0.106	0.535	1.112	0.796	1.553
PA (WHO)	inactive vs. Active	0.091	0.521	1.095	0.830	1.444
Well-being (WHO-5)	depressive vs. non-depressive	0.236	0.115	1.266	0.944	1.697

Abbreviations: OEC = online exercise class, BMI = body mass index, PA = physical activity and WHO = World Health Organization.

**Table 4:** Differences between users (n = 536) and non-users (n = 249) of OEC.

		OEC No Use	OEC Use	X^2^ _(785, 1)_	*p*-Value	ES
Sex	female (%)	25.9%	74.1%	35.672	<0.001	0.21
	male (%)	48.7%	51.3%			
Age (years)	≥50 (%)	35.5%	64.5%	3.902	0.048	0.07
	<50 (%)	28.9%	71.1%			
BMI (kg/m^2^)	≥30 (%)	43.9%	56.1%	6.275	0.012	0.09
	<30 (%)	30.3%	69.7%			
Education	<Bachelor (%)	35.3%	64.7%	2.015	0.156	0.05
	≥Bachelor (%)	30.2%	69.8%			
PA	inactive (%)	39.0%	61.0%	38.408	<0.001	0.22
	active (%)	17.2%	82.8%			
Well-being (WHO-5 pts.)	<50 (%)	30.9%	69.1%	0.196	0.658	0.02
	≥50 (%)	32.4%	67.6%			

Abbreviations: OEC = online exercise classes, ES = effect size, BMI = body mass index, PA = physical activity and WHO = World Health Organization.

**Table 5:** Frequency of the use of online exercise classes according to physical activity status.

Frequency of OEC Use		Inactive	Active	Total
Missing	counts	0	1	1
	%	0.0%	0.4%	0.2%
<1/week	counts	134	128	262
	%	51.1%	46.7%	48.9%
1–2/week	counts	96	92	188
	%	36.6%	33.6%	35.1%
≥3/week	counts	32	53	85
	%	12.2%	19.3%	15.9%
Total	counts	262	274	536
	%	100%	100%	100%

Abbreviation: OEC = online exercise class.

**Table 6:** Binary logistic regression to predict the use of online exercise classes.

Dependent:	OEC Use	β	*p*-Value	OR	95% CI	
n = 785					Lower	Upper
Sex	female vs. male	1.501	<0.001	4.486	3.316	6.069
Age	50 or older vs. younger	−0.043	0.782	0.958	0.707	1.298
BMI	obese vs. BMI < 30	−0.402	0.114	0.669	0.407	1.101
Education	non-academic vs. academic	−0.215	0.221	0.807	0.572	1.138
PA (WHO)	inactive vs. active	−0.671	<0.001	0.511	0.377	0.693
Well-being (WHO-5)	depressive vs. non-depressive	0.344	0.028	1.410	1.037	1.918

Abbreviations: OEC = online exercise class, BMI = body mass index, PA = physical activity and WHO = World Health Organization.

**Table 7:** Spearman correlations of opinions towards OECs.

Opinion	Spearman	Sex	Age	BMI	Education Level	PA (Active/Inactive)	Well-Being	OEC Use Frequency
Flexible time management	rho	0.107	0.043	0.080	−0.111	−0.158	−0.158	−0.294
	*p*-value	0.013	0.321	0.064	0.010	<0.001	<0.001	<0.001
Lacking interaction with exercisers	rho	−0.080	0.071	−0.059	0.070	0.053	0.135	0.129
	*p*-value	0.065	0.099	0.174	0.105	0.221	0.002	0.003
Lower motivation	rho	−0.131	0.101	−0.010	0.093	0.184	0.121	0.317
	*p*-value	0.002	0.019	0.820	0.032	<0.001	0.005	<0.001
Lacking instructor’s support	rho	−0.028	−0.029	−0.102	0.066	0.147	0.174	0.177
	*p*-value	0.518	0.509	0.018	0.130	0.001	<0.001	<0.001
Boringness	rho	−0.171	0.002	−0.079	0.021	0.187	0.179	0.420
	*p*-value	<0.001	0.972	0.069	0.632	<0.001	<0.001	<0.001

Abbreviations: OEC = online exercise class, PA = physical activity and BMI = body mass index.

## Data Availability

The data presented in this study are available on request from J.S.
